# Quality by Design Assisted Optimization of a Chiral Capillary Electrokinetic Chromatographic Method for the Separation of Amlodipine Enantiomers Using Maltodextrin as Chiral Selector

**DOI:** 10.3390/ph15030319

**Published:** 2022-03-07

**Authors:** Ratih Ratih, Hermann Wätzig, Matthias Oliver Stein, Sami El Deeb

**Affiliations:** 1 Institute of Medicinal and Pharmaceutical Chemistry, Technische Universität Braunschweig, 38106 Braunschweig, Germany; ratih_rath@staff.ubaya.ac.id (R.R.); h.waetzig@tu-braunschweig.de (H.W.); matthias.stein@tu-braunschweig.de (M.O.S.); 2 Department of Pharmaceutical Chemistry, Faculty of Pharmacy, University of Surabaya, Surabaya 60293, Indonesia; 3 Natural and Medical Sciences Research Center, University of Nizwa, P.O. Box 33, Birkat Al Mauz, Nizwa 616, Oman

**Keywords:** amlodipine, capillary electrophoresis, chiral capillary electrokinetic chromatography, design of experiment, D-optimal design, enantioseparation, quality by design, maltodextrin

## Abstract

Analytical-method development based on design of experiment has been applied for optimizing the enantioseparation of amlodipine by chiral capillary electrokinetic chromatography using maltodextrin as the chiral selector. The effect of different factors on the enantioresolution quality was screened. Three separation factors, namely maltodextrin concentration, pH of the background electrolyte and applied voltage were selected as independent variables. The number of experiments was reduced while maximizing the information content using design of experiment. Based on a full-quadratic design that included three variables on three levels, the total design space could be reduced to fifteen factor combinations using a D-optimal algorithm. The aim of the experiment was to find the optimal factor combinations with respect to resolution. The maltodextrin concentration (7.5–10% *w*/*v*) demonstrated the strongest effect on the resolution followed by pH (2–4) of the background electrolyte and the applied voltage (15–20 kV). An increase in the maltodextrin concentration was found to result in a greater stereoselectivity, represented by the higher resolution values (*R*_s_ ≥ 1.5). The separation conditions in the proposed method were feasible to be adjusted within the applied range with an acceptable resolution.

## 1. Introduction

Chiral separations using chiral stationary phases in liquid chromatography and buffer additives in capillary electrophoresis (CE) are the techniques of choice for analytical purposes. Chiral CE offers more simplicity in changing the employed chiral selector and adjusting its concentration in the background electrolyte (BGE) [1,2]. Since the chiral selector in the BGE acts as pseudostationary phase and the separation follows chromatograpic principles, the term chiral electrokinetic chromatography (*c*EKC) is used to describe chiral CE [3,4].

Over the years, cyclodextrines have become the most common chiral selectors. Due to its hydrophobic cavity, it shows chiral recognition toward various drug enantiomers [5,6,7]. Alternative chiral selectors are Maltodextrins (MDs). The hydrophobic properties of the inner helical structure lead to similar characteristics as the cyclodextrins’ cavity [8]. Maltodextrins are oligosaccharides which can be characterized by a dextrose equivalent (DE) value. The DE is defined by the extent of starch hydrolysis [8,9]. The sugar moeties contribute to the chiral recognition by forming hydrogen bonds, dipole–dipole and CH-π interactions. These interactions sterically complement their helical structure, which is expected to be the basis of the stereoselective behavior [8,10,11]. 

In previous studies, Tabani et al. reported that maltodextrin with low DE (4–7) has a higher degree of stereoselectivity compared to DE (13–17) and DE (16.5–19.5) [10]. Its high aqueous solubility and low absorbance in the UV region allow MD to be employed at relatively high additive concentrations. It is interesting to further investigate the reliable range of MD concentration in *c*EKC. However, the concentration of the chiral selector in the BGE is not the only factor which has influence on the separation in *c*EKC. The separation can be affected by other relevant factors such as the pH or the applied voltage [12]. Since a number of factors can influence the quality of an analytical measurement, many experimental trials may be necessary in method development. Thus, this process can be very laborious and time-consuming [1,13]. 

In order to reduce the number of experiments, systematic multivariate method optimization using design of experiment (DoE) is highly recommended [14]. The assessed response can be modeled using a multivariate linear equation. Usually, low-degree polynomials with interaction terms are used as model equations. Contour surface plots can be used to visualize how the response is affected by factors and to find combinations of optimal conditions. Optimal designs, such as D-optimal designs allows for the identification of the critical factors and an interaction between variables with maximal information and a minimum number of trials [15]. This approach reduces the analysis time and results in a more efficient experiment. 

Amlodipine (AML) is a calcium antagonist with a chiral center and marketed as a racemic drug. However, the eutomer, (*S*)-(−)-amlodipine is currently commercially available in some countries in Asia (e.g., China, India, Korea, Philiphines, Nepal) and Europe (e.g., Russia, Ukraine) [16]. A clinical study proved that the pharmacokinetic behavior of AML racemate and its single enantiomer is comparable [17]. Later, the efficacy and safety of racemic AML was studied on hypertensive patients [18]. Recently, Ermakov and Pashanova published a comparative study, providing an efficacy assessment of racemic AML vs. the (*S*)-(−)-enantiopure [19]. In addition, analytical studies on enantiopurity assays and impurity tests of amlodipine in pharmaceutical formulations have been performed [20,21]. The mentioned studies show that AML represents a chiral drug that is continuously monitored and clinically evaluated when administered as a racemate and/or a single-enantiomer [16]. Since the quality, safety, and efficacy of drugs are of critical importance, analytical methods, especially for drug enantiomers, are required.

Enantioseparation of AML using sulfonylbuthylether-β-cyclodextrin and polyethylene glycol 20,000 as dual chiral additives was performed using HPLC [22]. In 2016, Kannappan et al. identified the effect of factors on AML enantioresolution and analysis time using a cellulose-based HPLC column by employing Box–Behnken design [20]. Chiral selector screening on cyclodextrin derivatives and using an enantioseparation method optimization of AML in CE with an orthogonal experimental design were reported [23]. However, as an alternative potential chiral selector for AML [10,24], MD with DE (4–7) has not been systematically optimized to identify factors affecting resolutions. 

This study proposes a systematic *c*EKC method optimization to find the optimal factor combinations for the resolution of AML enantiomers. The MD concentration, the BGE pH, and applied voltage were selected as the independent variables and combined using a D-optimal design. This approach provides efficient method optimization with a minimum number of combinations instead of a one-factor-at-a-time experiment.

## 2. Results 

### 2.1. Effect of Separation Factors on Resolution

The full factorial design with three separation factors on three levels was reduced to 15 combinations using a D-optimal algorithm. All investigated factor combinations and the corresponding measurement results are listed in Table 1. 

### 2.2. Enantioseparation Profiles

**Figure 1:**
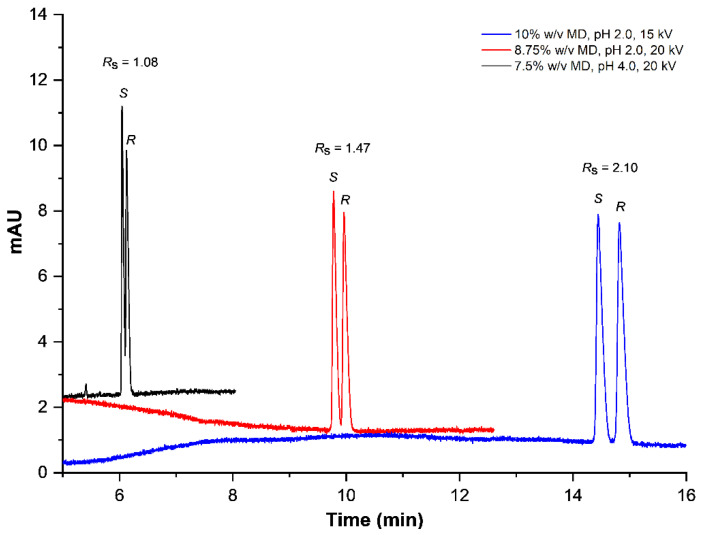
Representative enantioseparation of AML at the shortest and longest analysis time.

In the 15 combinations, the obtained resolution varied between *R*_s_ = 1.07 and *R*_s_ = 2.10, with total analysis times from 7 to 20 min. The (*S*)-(−)-enantiomer of AML eluted as the first peak followed by the (*R*)-(+)-enantiomer. Electropherograms correspond to the minimum resolution at the shortest analysis time and the maximum resolution at the longest analysis time, as depicted in Figure 1. 

The highest resolution, as depicted by the blue electropherogram, is *R*_s_ = 2.10. In contrast the black electropherogram shows the lowest resolution, with *R*_s_ = 1.07. The red electropherogram represents another resolution profile between the highest and the lowest *R*_s_ values. The migration order of AML enantiomers was found to be the (*S*)-(−)-enantiomer followed by the (*R*)-(+)-enantiomer. Based on the migration order, it can be deduced that (*R*)-(+)-amlodipine possesses stronger binding toward MD. The separation profiles show that the resolution increases with an increasing MD concentration. Next to its effect on the chemical equilibrium of the binding reaction, it is expected that higher concentrations of MD might increase BGE viscosity. Consequently, the migration velocity slows down, which elongates the analysis time. Similar effects on the analysis time can be expected when lowering the pH. Since a low pH decreases the charge density on the inner capillary wall, the EOF is reduced. Together with a low voltage, these two effects cumulatively prolong the total analysis and thus increase the time of interaction. In summary, the measurements show a general positive correlation of the MD concentration with both the analysis time and the resolution. The other two experimental factors demonstrate the opposite correlation. However, to find an optimum between a short analysis time and a favorable resolution, a one-factor-at-a-time experiment is not the best option. Since all these effects might interact with one another, a systematic investigation using DoE is recommended.

### 2.3. The Most Affecting Factors and Predicted Responses

Factor combinations and the obtained resolution were further evaluated according to polynomial (quadratic) regression model, see Equation (1). All main effects (voltage (U), MD concentration (MD), pH), single interaction between pH and U as well as quadratic effects of U and pH (U^2^ and pH^2^) demonstrated significant effects on the resolution. The same factors, aside from the quadratic term of the voltage, were found to be relevant for the analysis time prediction. The predicted resolution (${\hat{R}}_{S}$) or analysis time (${\hat{t}}_{m}$) can be calculated using the coefficients as listed in Table 2. All non-significant coefficients are considered to be 0 for the prediction.

(1) $${\hat{R}}_{S}=\beta_{0}+\overset{N}{\underset{i=1}{\sum }}\beta_{i}x_{i}+\overset{N}{\underset{i=1}{\sum }}\beta_{ii}x_{i}{}^{2}+\overset{N-1}{\underset{i=1}{\sum }}\overset{N}{\underset{j=i+1}{\sum }}\beta_{ij}x_{i}x_{j}$$

## 3. Discussion

### 3.1. Rationals of the Factor Selection and Definition of the Design Space

A systematic maltodextrin-based *c*EKC method optimization involving three factors on three different levels was developed for AML enantiomer separation. MD DE (4–7) at concentrations of 7.5–10% *w*/*v* was employed as the chiral selector, the BGE was adjusted to pH values between 2.0 and 4.0 and the applied voltage ranged from 15 to 20 kV. Performing *c*EKC for AML separation at a voltage lower than 15 kV (263 V/cm) for a 57 cm capillary was not recommended due to a decrease in the resolution related to the CE efficiency [24]. Thus, a voltage range of 15–20 kV (330–440 V/cm) for a 45.5 cm capillary was employed in this study.

Nojavan et al. reported a one-dimensional analysis of the MD concentration effect on the resolution using 5–20% *w*/*v* MD. They demonstrated that baseline separation can be achieved with MD concentrations of about 10% *w*/*v* [24]. However, since an increase in MD concentration prolongs the analysis time, the feasibility of performing baseline separation wen utilizing a maximum of 10% *w*/*v* MD was investigated. To perform the separation in a reasonable time of analysis, BGE pH in a range of 2.0–4.0 was selected as an additional factor. In order to perform a quadratic regression, the selected range of each factor was employed in its minimum, middle, and maximum levels.

### 3.2. Evaluation of the Factor Effects on the Resolution

**Figure 2:**
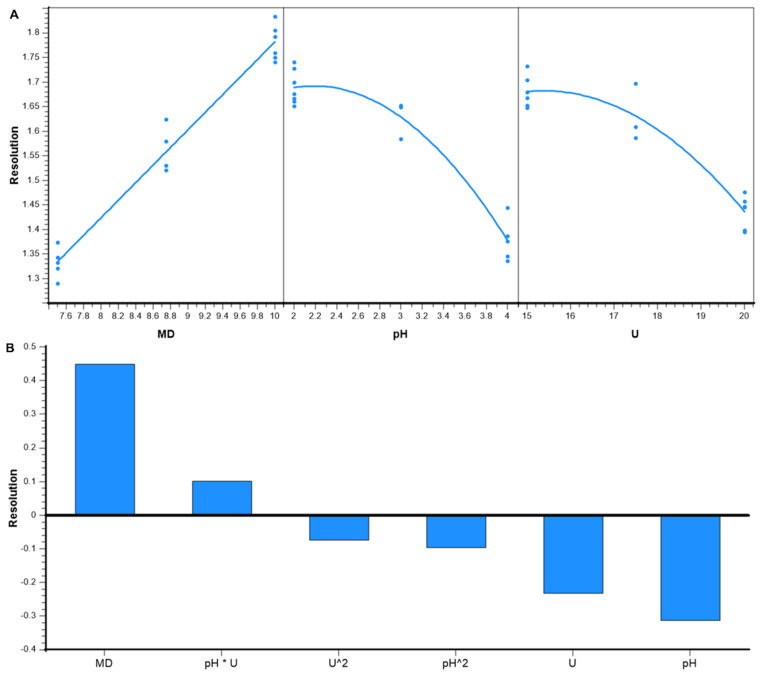
The adjusted response graph of resolution (**A**) and Pareto chart of the effects of variables on resolution (**B**). MD: maltodextrin concentration (% *w*/*v*); U: voltage (kV).

An adjusted response graph and a Pareto chart were used to further evaluate of the effects on the obtained resolution. Figure 2 summarizes the main effects depicted as an adjusted response graph (Figure 2A) and as Pareto chart (Figure 2B). The adjusted response graph shows the effect of a single factor, while the effects of the others is averaged out. The MD concentration showed positive linear relationships with a shift of resolution from about *R*_s_ = 1.3 (MD low) to *R*_s_ = 1.8 (MD high). In contrast, both pH and voltage showed negatively curved relationships, which means that the model must be at least quadratic. A resolution of about *R*_s_ = 1.7 (low pH), *R*_s_ = 1.6 (middle pH), and *R*_s_ = 1.3 (high pH) were shown at the three investigated pH levels. A similar range of resolution was found for the voltage levels between *R*_s_ = 1.7 (low U) and *R*_s_ = 1.4 (high U).

The Pareto chart indicates the strength of every term in a comparable bar chart. Positive and negative bars indicate whether a term correlates positively or negatively with the predicted resolution. Since the variable domains were coded to be in the range from −1 to 1 the absolute height is a comparable measure of the term’s strength. The MD concentration was found to be the variable with the strongest effect on the resolution, followed by pH, applied voltage, the interaction between pH and voltage, and the quadratic effects (U^2^ and pH^2^). The MD concentration and the interaction between pH and the U are positively correlated, represented by bars pointing upward. On the other hand, the pH, quadratic pH, voltage, and quadratic voltage are negatively correlated. In summary this means, that the highest resolution is expected using a combination of high MD concentration and both low pH and voltage. 

**Figure 3:**
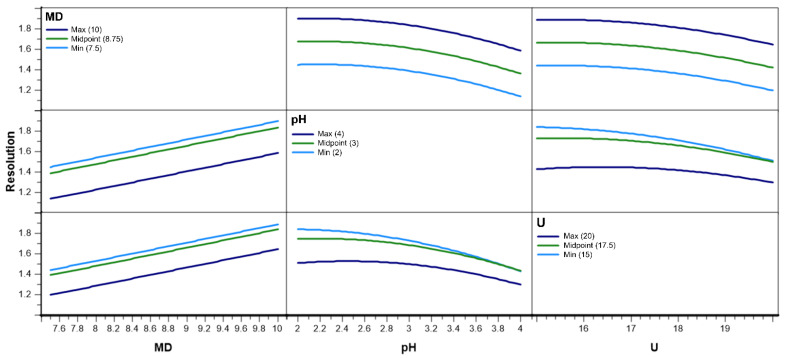
Interaction graph for resolution at low, middle, and high levels. MD: maltodextrin concentration (% *w*/*v*); U: voltage (kV).

The magnitude of the factor interactions was evaluated using the regression coefficients in Equation (1) (see Table 2) and visually by interaction graphs. The interactions graphs for resolution are depicted in Figure 3. 

The interaction graphs shown in Figure 3 consist of six panels, where every panel represents an interaction between two factors. Every interaction is displayed from two different perspectives. These plots are to be interpreted in the following way. Every panel shows the change of the predicted resolution for one factor on a continuous scale, while another is given on three different fixed levels. The line colors represent the levels of the fixed interaction partner. Here, the light blue stands for low, green for middle, and dark blue for high. For instance, the resolution rises linearly with an increasing MD concentration, as shown in the lower left panel. The three different curves represent the change of resolution when the voltage is 20 kV (dark blue line), 17.5 kV (385 V/cm) for a 45.5 cm capillary (green line) and 15 kV (light blue line).

If there were to be an interaction between these two factors, then the level of U would affect the course of the resolution curves differently on different levels. In other words, the three curves would not be parallel. The typical non-parallel interaction graphs can be seen in the lower center and in the right center panels. Both panels depict the interaction between pH and U. Since the light blue line and the green line in these graphs are not parallel, the factors U and pH show an interaction. This finding is in accordance with the coefficient table in Table 2, where the interaction term between those two variables was found to be the only significant one.

### 3.3. Prediction of Resolution and Optimal Experimental Conditions

**Figure 4:**
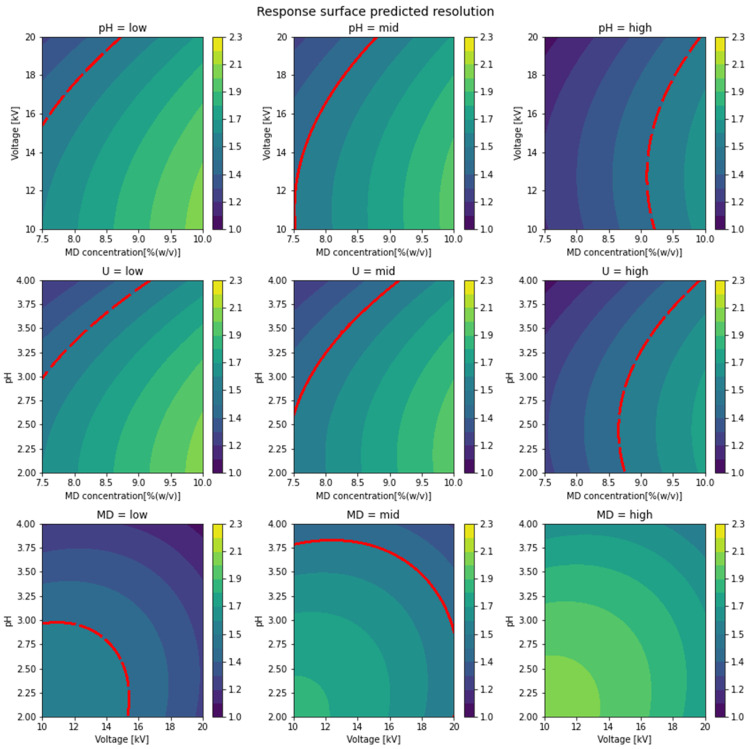
Contour plot of the predicted *R*_s_ at the respective combinations.

The predicted resolution shown as a response to contour plots of all three factors is depicted in Figure 4. Since only two of the three parameters can be shown in one graph simultaneously, the continuous variable space of two factors each is shown while the third factor is fixed on a defined level. Using information depicted in these following graphs, it is possible to find all factor combinations which lead the optimal or desired resolution. 

The predicted resolution is indicated by the color code. The red curved line represents the border to the obtained baseline resolution ${\hat{R}}_{S}$ ≥ 1.5. When using any factor combination below the curved red line, achieving the desired resolution can be expected. For instance, a higher *R*_s_ than 1.5 can be expected if one of the non-blue factor combinations of the central panel in Figure 4 is chosen when the voltage is 17.5 kV (385 V/cm)*^b^ for a 45.5 cm capillary. The lower right panel is a special case. The contour plot is presented without a curved red line, which means that every possible combination of U and pH lead to ${\hat{R}}_{S}$ ≥ 1.5 when the MD concentration is 10% *w*/*v*. In contrast, the lower left panel, where the MD concentration is 7.5% *w*/*v*, shows just a narrow area of high-resolution factor combinations. Overall, this underlines the high influence of the MD concentration. The depicted contour plots can be used to identify critical or optimal factor combinations, which result in an analysis with the desired resolution, especially in combination with the predicted total analysis time (data not shown). This analysis can be very helpful to find the best compromise between analysis time and resolution.

Baseline resolution of ${\hat{R}}_{S}$= 1.50 ± 0.17 and total analysis time (migration time of the second peak) of ${\hat{t}}_{m}$= 8.16 ± 1.46 min were predicted with a confident interval 95% using separation factors of MD 10% *w*/*v* (high), pH 4.0 (high), and voltage 20 kV (440 V/cm) for a 45.5 cm capillary (high). At the respective factor combination, the mean of six injections from the experimental measurement showed *R*_s_ = 1.47 ± 0.036 within an analysis time of *t*_m_ =7.8 ± 0.44 min. The experimental measurements were found to be in close agreement with the predicted values.

### 3.4. Method Robustness

The proposed method was developed in 2018 and verified in 2022 using different series of CE instruments with adjustments to ensure identical CE conditions. The method robustness was evaluated by comparing the performance of amlodipine enantioseparation in three CE instruments, as listed in Table 3.

Separation factors of MD 10% *w*/*v* (high), pH 4.0 (high), and voltage 20.7 kV (440 V/cm)*^c^ ≈ 20 kV (440 V/cm)*^b^ (high), resulted in *R*_s_ = 1.22–1.61 within analysis times of *t*_m_ = 7.802–9.583 min. Additional evaluation at the center point combination using separation factors of MD 8.75% *w*/*v* (mid), pH 3.0 (mid), and voltage equal to 18.1 kV (385 V/cm)*^c^ ≈ 17.5 kV (385 V/cm)*^b^ (mid) resulted in *R*_s_ = 1.39–1.69 within the analysis times of *t*_m_ = 12.335–12.763 min. The combination of separation factors MD 10% *w*/*v* (high), pH 2.0 (low), and voltage 15.5 kV (330 V/cm)*^c^ ≈ 15 kV (330 V/cm) for a 45.5 cm capillary (low), resulted in *R*_s_ = 1.94–2.14 within analysis times of *t*_m_ = 17.829–20.606 min.

The *R*_s_ values, detected by instrument C (PrinceCE Next 800), at separation conditions I and II, were less than ${\hat{R}}_{S}$. The small difference between experimental *R*_s_ and ${\hat{R}}_{S}$ might occur due to the fact that Cornerstone’s prediction was simulated using the initial data performed using PrinCE CEC-760 system. Overall, baseline enantioseparations obtained by all three CE instruments were close to the ${\hat{R}}_{S}$ and ${\hat{t}}_{m}$ at the respective separation conditions. These results showed the method robustness using three different instruments. 

### 3.5. Method Application

The selected enantioseparation method was applied to amlodipine identification and determination in tablet matrices. 

#### 3.5.1. Enantiomers Identification

**Figure 5:**
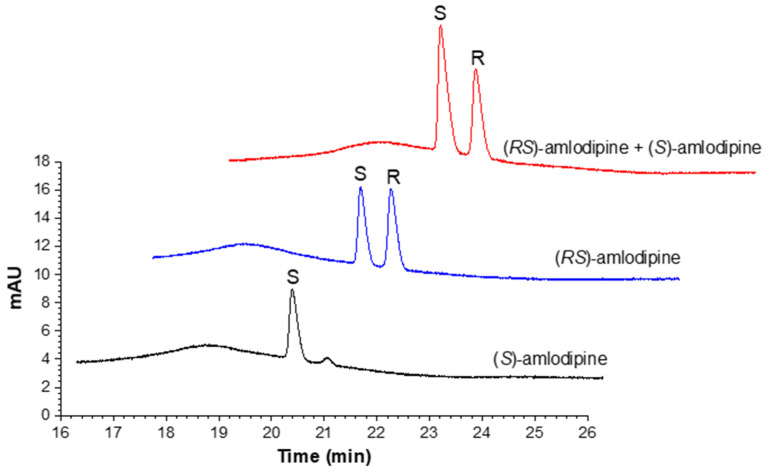
Enantioseparation profiles of amlodipine at the experimental condition MD 10% *w*/*v* (high), pH 2.0 (low), and voltage 15 kV (330 V/cm) for a 45.5 cm capillary (low). Peak identification shows that the migration order of amlodipine is the (*S*)-enantiomer followed by the (*R*)-enantiomer.

The migration order of amlodipine enantiomers was identified using (*S*)-amlodipine as a single compound and standard addition of (*S*)-amlodipine into (*RS*)-amlodipine. The stock solutions of (*S*)-amlodipine and (*RS*)-amlodipine were prepared in MeOH. (*RS*)-amlodipine (240 µg/mL), (*S*)-amlodipine (120 µg/mL), and a mixture of (*RS*)-amlodipine and (*S*)-amlodipine (2:1) in 100 mM phosphate buffer pH 2.0 were used as the injected samples as depicted in Figure 5.

#### 3.5.2. Enantiomeric Ratio

The determination of the enantiomeric ratio was performed using standard samples of (*S*)-amlodipine, (*RS*)-amlodipine, and a standard addition (a mixture of (*RS*)-amlodipine and (*S*)-amlodipine (2:1)) as listed in Table 4. The standard samples were prepared as described in Section 3.5.1.

#### 3.5.3. Enantiomers Determination

The selected separation method condition was evaluated based on several parameters prior to sample analysis as listed in Table 5.

**Figure 6:**
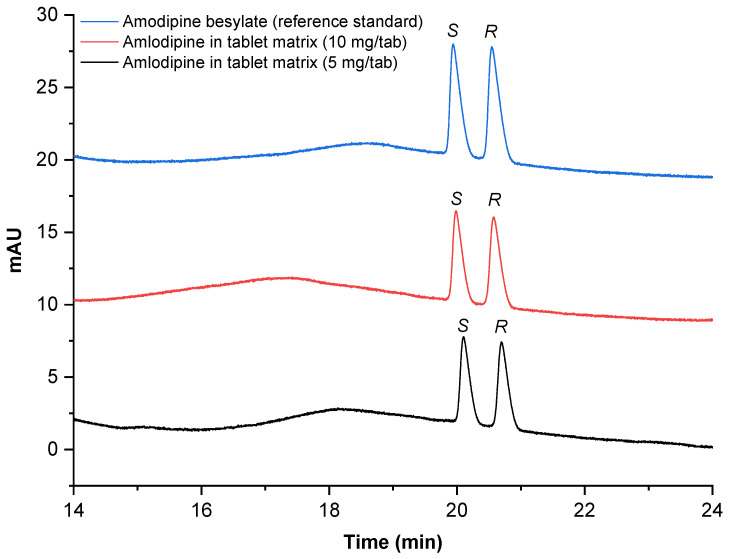
Enantioseparation profile of amlodipine in tablet matrices at the experimental condition MD 10% *w*/*v* (high), pH 2.0 (low), and voltage 15 kV (330 V/cm) for a 45.5 cm capillary (low).

Amlodipine determination in tablet matrices using two sample strengths of 5 mg/tablet and 10 mg/tablet is listed in Table 6. The amlodipine content and recovery were calculated based on the (*S*)-(−)-enantiomer which is pharmacologically the more active enantiomer than its antipode. This approach was conducted as a preliminary study for the separation-method application to amlodipine determination in tablet matrices. Thus, simplified sample analysis in triplicate injections was performed instead of triplicate preparations in an ideal pharmaceutical analysis. The enantioseparation profiles of amlodipine in tablet matrices are depicted in Figure 6. 

## 4. Materials and Methods

### 4.1. Materials

Maltodextrin (DE 4–7), sodium dihydrogen phosphate (NaH_2_PO_4_), sodium hydroxide (NaOH), ortho-phosphoric acid (H_3_PO_4_, 85%), (*RS*)-amlodipine (as amlodipine besylate was acquired from Sigma-Aldrich Chemie GmbH (Steinheim, Germany), enantiopure (*S*)-(−)-amlodipine from Biozol Diagnostica Vertrieb GmbH (Munich, Germany). Water was purified using Arium^®^ pro UF/VF-Sartopore 0.2 µm water purification system from Sartorius Weighing Technology GmbH (Göttingen, Germany). The phosphate buffer was prepared using 100 mM sodium dihydrogen phosphate to reach the final pH 2.0–4.0 of 1 L buffer solution. Solutions of 1 M NaOH, and 0.1 M NaOH were prepared in ultrapure water. All the solutions were filtered using nylon membrane 0.22 µM pore size from Rotilabo^®^-syringe filter, Carl Roth GmbH (Karlsruhe, Germany) prior the analysis.

The background electrolyte was prepared with the addition of MD at various concentrations (7.5–10% *w*/*v*) into 100 mM phosphate buffer (pH 2.0–4.0). A stock solution of (*RS*)-amlodipine was prepared in MeOH at a concentration of 1 mg/mL. A certain volume of the stock solution was dissolved in 100 mM phosphate buffer (pH 2.0–4.0) to the final concentration of 300 µg/mL and used as the injected sample.

The enantiomers were determined using a calibration curve prepared from the stock solution of (*RS*)-amlodipine and diluted in 100 mM phosphate buffer pH 2.0 to five final concentrations (180–600 µg/mL). Two commercially available amlodipine tablets (5 mg/tablet and 10 mg/tablet) were selected as the samples. Tablets (10) from each strength were weighed and ground into fine powder. Each sample, which was equal to the average weight of one tablet, was dissolved in MeOH with 15 min ultrasonication at room temperature. Samples were filtered using a 0.22 µm filter membrane and diluted in a 100 mM phosphate buffer of pH 2.0 to certain concentrations (≈230–270 µg/mL amlodipine).

### 4.2. CE Instrumentation

The enantioseparation study was performed with a PrinCE CEC-760 system (Prince Technologies, Emmen, The Netherlands) using a diode array UV-Vis detector (190–600 nm). The DAx 3D software (version 9.0) was used for instrumental control, data acquisition, and data analysis. The robustness of the method was verified using the second instrument unit of a PrinCE CEC-760 system (DAx 3D software) and a PrinCE Next-800 series (Clarity software) from Prince Technologies, Emmen, The Netherlands. Bare fused-silica capillaries from were kindly provided by Polymicro Technologies (Phoenix, AZ, USA) with 50 μm inner and 360 μm outer diameters, 45.5 cm total length and 37 cm effective length were used throughout the study. The sample rack and capillary oven were set at 25 °C.

### 4.3. D-Optimal Design

The D-optimal design of three factors with three levels at maltodextrin concentration (7.5% *w*/*v*, 8.75% *w*/*v*, 10% *w*/*v*), applied voltage (15 kV, 17.5 kV, 20 kV), and pH (2.0, 3.0, 4.0) was derived by DoE software Cornerstone 7.0, camLine Holding AG (Peterhausen, Germany). The model considers the effect of the selected factors on the enantioseparation that possesses restraint values for the design space, as listed in Table 7.

The D-optimal design of a constant, three main effects, three quadratic effects, and three single interactions, with five extra points for degrees of freedom (see Table 1).

### 4.4. Experimental and Statistical Data Evaluation

The enantiomeric resolution (*R*_s_) was calculated according to the standard expression based on the peak full-width at half-maximum by DAx 3D software, as depicted in Equation (2).

(2) $$R_{s}=1.18\times \frac{t_{2}-t_{1}}{\left(W_{1}+W_{2}\right)}$$

Here, the migration times of enantiomer one and enantiomer one are $t_{1}$ and $t_{2}$ along with the full widths at the half-maximum of $W_{1}$ and $W_{2}$, respectively. The coefficients for the prediction of the resolution were computed using partial least square regression of the multivariate data to the model given in Equation (1). Regression coefficients were regarded as significant when *p* < 0.1.

## 5. Conclusions

Systematic method optimization with a design of experiment was applied using D-optimal design to investigate the combinations of separation factors in maltodextrin-based *c*EKC. The separation factors at a high MD concentration, low pH value, and low applied voltage provided the highest resolution of AML enantiomers of about *R*_s_ = 2.10 within 20 min. The baseline resolution of ${\hat{R}}_{S}$ = 1.50 ± 0.17 in the shortest possible time was predicted using the separation factor combination of high MD concentration, high pH, and high applied voltage. The predicted total analysis time of the proposed experimental setup was ${\hat{t}}_{m}$ = 8.16 ± 1.46 min. Compared to other reported studies, this proposed *c*EKC method offers the advantages of a better baseline resolution in a shorter migration time. The separation factors of MD concentration showed the strongest effect on the resolution followed by the pH of the BGE and the applied voltage. The most affecting factors were defined to guarantee an excellent method robustness. The identification and determination of amlodipine in tablet matrices with acceptable recoveries showed the applicability of the optimized method at the selected separation factor combinations.

## Figures and Tables

**Table 1:** The effect of factor combinations on the resolution of AML enantiomers.

Factor Combinations	Voltage	MD	pH	*R*_s_	SD	*t*_2_	SD
	kV	% *w*/*v*				Min	
1	20	10	2.0	1.73	0.03	12.15	0.47
2	15	8.75	2.0	1.80	0.02	16.42	0.39
3	15	10	4.0	1.61	0.05	11.37	0.55
4	15	10	2.0	2.10	0.06	17.83	0.64
5	15	10	3.0	1.96	0.08	16.06	0.58
6	17.5	10	2.0	1.93	0.02	14.73	0.32
7	17.5	7.5	3.0	1.40	0.03	10.46	0.37
8	15	7.5	2.0	1.59	0.04	15.58	0.80
9	20	7.5	2.0	1.31	0.02	10.28	0.36
10	20	10	4.0	1.47	0.03	7.80	0.42
11	20	8.75	3.0	1.50	0.02	10.19	0.02
12	17.5	8.75	4.0	1.49	0.08	8.88	0.77
13	20	8.75	2.0	1.46	0.01	10.27	0.17
14	15	7.5	4.0	1.19	0.04	8.62	0.37
15	20	7.5	4.0	1.07	0.04	6.04	0.31

Each *R*_s_ value is an average from 6 injections; *t*_2_: second eluted peak.

**Table 2:** Regression coefficients of the predicted quadratic polynomial for the response variable.

Term	Resolution			Analysis Time		
	Coefficient	SE	Sig.	Coefficient	SE	Sig.
Constant	1.67	0.0338	3.07·10^−11^	12.2	0.256	3.88·10^−12^
Voltage (U)	−0.115	0.0137	3.10·10^−5^	−2.22	0.131	3.85·10^−8^
Concentration (MD)	0.224	0.0142	2.65·10^−7^	1.18	0.135	1.08·10^−5^
pH	−0.156	0.0137	3.26·10^−6^	−2.59	0.1303	9.57·10^−9^
pH × U	0.0499	0.0150	0.0104	0.654	0.143	0.00132
U^2^	−0.0720	0.0306	0.0464	---	---	---
pH^2^	−0.0954	0.0306	0.0143	−1.11	0.288	0.00390
Adj. R^2^	0.974			0.984		
RMSE	0.0466			0.444		

**Table 3:** Robustness verification of the enantioseparation method.

Separation Condition	Instrument (Year)	L_t_/L_eff_ (cm)	E (V/cm)	U (kV)	MD (% *w*/*v*)	pH	Experiment *		Predicted	
							*R*_s_ ± SD	$t_{m}$ ± SD	${\hat{R}}_{S}$ ± SD	${\hat{t}}_{m}$ ± SD
I	Instrument A (2018)	45.5/37	440	20	10	4	1.50 ± 0.03	7.802 ± 0.422	1.50 ± 0.17	8.159 ± 1.464
	Instrument B (2022)	45.5/37	440	20	10	4	1.61 ± 0.11	9.213 ± 0.653		
	Instrument C (2022)	47/37	440	20.7	10	4	1.22 ± 0.04	9.583 ± 0.329		
II	Instrument A (2018)	45.5/37	385	17.5	8.75	3	-- **	-- **	1.67 ± 0.17	12.236 ± 1.153
	Instrument B (2022)	45.5/37	385	17.5	8.75	3	1.69 ± 0.07	12.763 ± 0.790		
	Instrument C (2022)	47/37	385	18.1	8.75	3	1.39 ± 0.04	12.335 ± 0.150		
III	Instrument A (2018)	45.5/37	330	15	10	2	2.10 ± 0.06	17.829 ± 0.641	2.05 ± 0.14	17.783 ± 1.191
	Instrument B (2022)	45.5/37	330	15	10	2	2.14 ± 0.07	20.606 ± 0.092		
	Instrument C (2022)	47/37	330	15.5	10	2	1.94 ± 0.03	18.113 ± 0.085		

Instrument A: PrinCE CEC-760 system (unit 1); Instrument B: PrinCE CEC-760 system (unit 2); Instrument C: PrinceCE Next 800 series. I: MD 10% *w*/*v* (high), pH 4.0 (high), and voltage 20.7 kV (440 V/cm)*^c^ ≈ 20 kV (440 V/cm)*^b^ (high). II: MD 8.75% *w*/*v* (mid), pH 3.0 (mid), and voltage 18.1 kV (385 V/cm)*^c^ ≈ 17.5 kV (385 V/cm)*^b^ (mid). III: MD 10% *w*/*v* (high), pH 2.0 (low), and voltage 15.5 kV (330 V/cm)*^c^ ≈ 15 kV (330 V/cm)*^b^ (low). * Experiment: each condition 6 injections. ** The mid (center point) experiment condition was not conducted in 2018.

**Table 4:** Determination of enantiomeric ratio.

Analyte	Ratio (%)	
	*S*	*R*
(*S*)-amlodipine	91.8 ± 0.9	8.2 ± 0.9 *
(*RS*)-amlodipine	50.1 ± 0.1	49.9 ± 0.1
Standard addition **	65.2 ± 0.4	34.8 ± 0.4

Experiment in triplicate injections. * assign as enantiomeric impurity. ** mixture of (*RS*)-amlodipine and (*S*)-amlodipine at a final concentration (2:1).

**Table 5:** Method evaluation.

Parameter	*S*	*R*
Range (µg/mL)	180–600	180–600
Linearity	0.9970	0.9842
LOD * (µg/mL)	30	69
LOQ ** (µg/mL)	91	209
Accuracy (%)	90–96	104–111
Precision *** (% RSD)	0.9	1.8

The values correspond to the analyte concentration in a racemate. * 3.3 RMSE/slope; ** 10 RMSE/slope; RMSE: root mean square error. *** Precision of enantiomeric ratio with a standard addition (2:1) (*n* = 6).

**Table 6:** Amlodipine determination in tablet matrices.

	A	B
Content (mg/tablet) *	5.32 ± 0.02	10.18 ± 0.13
Recovery (%) **	106.4 ± 0.4	101.8 ± 1.3

The determination correspond to the first eluted peak. Experiment in triplicate injections; A: amlodipine 5 mg/tablet; B: amlodipine 10 mg/tablet. * Tablet weight (mg) ($\bar{x}$ ± SD, *n* = 10): 220.1 ± 1.7 (A) and 223.1 ± 1.7 (B). ** Based on amlodipine strength in the label claim (product specification).

**Table 7:** The experimental domains of the D-optimal design.

Factors	Code	Levels		
		−1 (Low)	0 (Mid)	+1 (High)
Voltage (kV)	U	15	17.5	20
MD conc. (% *w*/*v*)	MD	7.5	8.75	10
pH	pH	2.0	3.0	4.0

## Data Availability

Data is contained within the article and Supplementary Materials.

## Supplementary Materials

The following supporting information can be downloaded at: https://www.mdpi.com/article/10.3390/ph15030319/s1, Figure S1: Enantioseparation profiles of amlodipine at the experimental condition MD 10% *w*/*v* 21 (high), pH 2.0 (low), and voltage 15 kV (330 V/cm)*^b^ (low); Figure S2: Enantioseparation profile of amlodipine in tablet matrices at the experimental condi-62 tion MD 10% *w*/*v* (high), pH 2.0 (low), and voltage 15 kV (330 V/cm)*^b^ (low), Table S1: Method validation, Table S2: Amlodipine determination in tablet matrices, Table S3: Determination of enantiomeric fraction.
