# Supplementary material for: Quality by Design Assisted Optimization of a Chiral Capillary Electrokinetic Chromatographic Method for the Separation of Amlodipine Enantiomers Using Maltodextrin as Chiral Selector
Source: Pharmaceuticals (Basel). 2022 Mar 7;15(3):319. doi: 10.3390/ph15030319 (PMC8955793; doi:10.3390/ph15030319)
Supplement: Supplementary file 1 [file pharmaceuticals-15-00319-s001.zip › pharmaceuticals-1475976-supplementary.pdf]

pharmaceuticals

Supporting material

Quality by design assisted optimization of a chiral capillary electrokinetic chromatographic method for the separation of amlodipine enantiomers using maltodextrin as chiral selector

Ratih Ratih [1,2,‡], Hermann Wätzig [1], Matthias Oliver Stein [1,‡], and Sami El Deeb [1,3*]

[1] Institute of Medicinal and Pharmaceutical Chemistry, Technische Universität Braunschweig, 38106 Braunschweig, Germany; ratih.ratih@tu-braunschweig.de or ratih_rath@staff.ubaya.ac.id (R.R.); h.waetzig@tu-braunschweig.de (H.W.); matthias.stein@tu-braunschweig.de (M.O.S.)
[2] Department of Pharmaceutical Chemistry, Faculty of Pharmacy, University of Surabaya, Surabaya 60293, Indonesia
[3] Natural and Medical Sciences Research Center, University of Nizwa, P.O. Box 33, Birkat Al Mauz, Nizwa 616, Sultanate of Oman

‡ These authors contributed equally to this work
* Correspondence: s.eldeeb@tu-braunschweig.de; Tel.: +49-531-391-7301 (S.E.)

Citation: Ratih, R.; Wätzig, H.; Stein, M.O.; El Deeb, S.; Quality by design assisted optimization of a chiral capillary electrokinetic chromatographic method for the separation of amlodipine enantiomers using maltodextrin as chiral selector. *Pharmaceuticals* **2022**, *15*, 319. https://doi.org/10.3390/ph15030319

Received: 7 November 2021
Accepted: 1 March 2022
Published: 7 March 2022

Publisher's Note: MDPI stays neutral with regard to jurisdictional claims in published maps and institutional affiliations.

Copyright: © 2022 by the authors. Licensee MDPI, Basel, Switzerland. This article is an open access article distributed under the terms and conditions of the Creative Commons Attribution (CC BY) license (https://creativecommons.org/licenses/by/4.0/).

I. Enantiomers identification

The migration order of amlodipine enantiomers was identified using (*S*)-amlodipine as a single compound and standard addition of (*S*)-amlodipine into (*RS*)-amlodipine. The stock solutions of (*S*)-amlodipine and (*RS*)-amlodipine were prepared in MeOH. (*RS*)-amlodipine (240 µg/mL), (*S*)-amlodipine (120 µg/mL), and a mixture of (*RS*)-amlodipine and (*S*)-amlodipine (2:1) in 100 mM phosphate buffer were used as the injected samples.

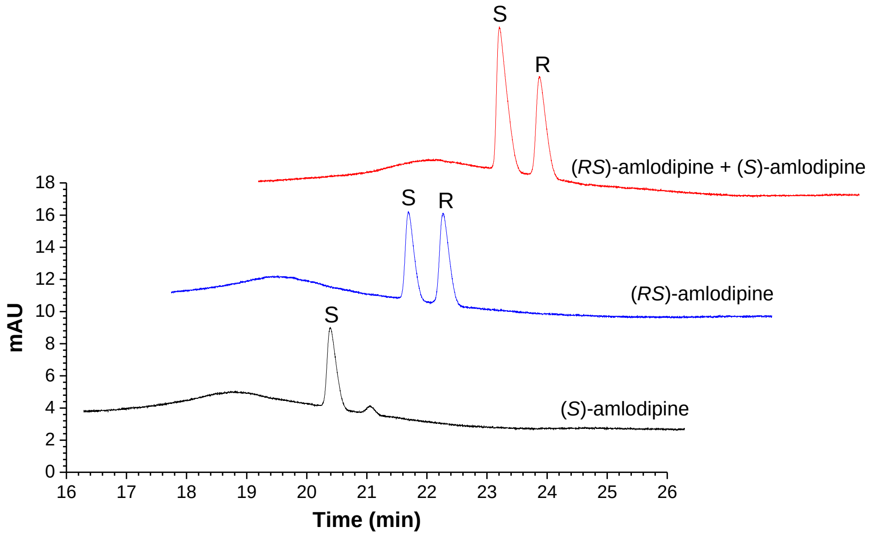

Figure S1. Enantioseparation profiles of amlodipine at the experimental condition MD 10% *w/v* (high), pH 2.0 (low), and voltage 15 kV (330 V/cm)[*b] (low). Peak identification shows that the migration order of amlodipine is (*S*)-enantiomer followed by the (*R*)-enantiomer.

[*]E of the capillary 45.5 cm[*b]

II. Enantiomers determination

A. Preparation

Calibration curve

Stock solution of (*RS*)-amlodipine was prepared in MeOH and diluted in 100 mM phosphate buffer to 5 final concentrations (180-600 µg/mL).

Tablet samples

Two commercially available amlodipine tablets (5 mg/tablet and 10 mg/tablet) were selected as the samples. Tablets (10) from each strength were weighed and ground into fine powder. Each sample, which was equal to the average weight of one tablet, was dissolved in MeOH with 15 min ultrasonication at room temperature. Samples were filtered using a 0.22 µm filter membrane and diluted in 100 mM phosphate buffer to a certain concentration (≈ 230 µg/mL - 270 µg/mL amlodipine).

B. Analysis

Table S1. Method validation

Parameter	*S*	*R*
Range (µg/mL)	180 - 600	180 - 600
Linearity	0.9970	0.9842
LOD* (µg/mL)	30	69
LOQ** (µg/mL)	91	209
Accuracy (%)	90 -96	104-111
Precision*** (% RSD)	0.9	1.8

The values correspond to the analyte concentration in a racemic mixture.

*3.3 RMSE/slope; **10 RMSE/slope; RMSE: root mean square error.

***Precision of enantiomer fraction with a standard addition (2:1) (n = 6).

Table S2. Amlodipine determination in tablet matrices

	A	B
Content (mg/tablet)*	5.32 ± 0.02	10.18 ± 0.13
Recovery (%)**	106.4 ± 0.4	101.8 ± 1.3

The determination correspond to the first eluted peak.

Experiment in triplicate injections; A: amlodipine 5 mg/tablet; B: amlodipine 10 mg/tablet

*Tablet weight (mg) ($\bar{x}$ ± SD, n = 10): 220.1 ± 1.7 (A) and 223.1 ± 1.7 (B)

**Based on amlodipine strength in the label claim (product specification).

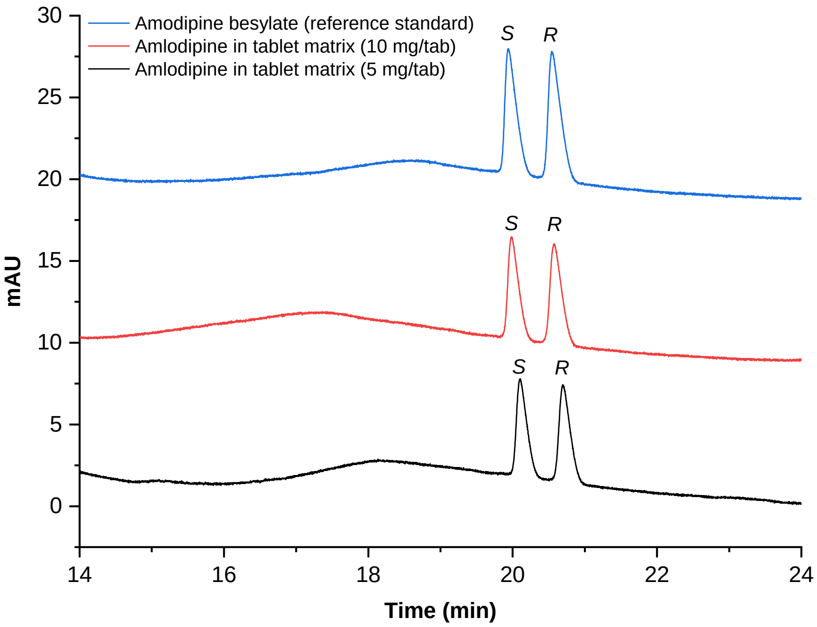

Figure S2. Enantioseparation profile of amlodipine in tablet matrices at the experimental condition MD 10% *w*/*v* (high), pH 2.0 (low), and voltage 15 kV (330 V/cm)*b (low).

*E of the capillary 45.5 cm*b

III. Enantiomeric fraction

Determination of enantiomeric fraction was performed using (*S*)-amlodipine, (*RS*)-amlodipine, and a standard addition [a mixture of (*RS*)-amlodipine and (*S*)-amlodipine (2:1)]. The samples were prepared as described in section I.

Table S3. Determination of enantiomeric fraction

Analyte	Fraction (%)	
	S	*R*
(*S*)-amlodipine	91.8 ± 0.9	8.2 ± 0.9*
(*RS*)-amlodipine	50.1 ± 0.1	49.9 ± 0.1
Standard addition**	65.2 ± 0.4	34.8 ± 0.4

Experiment in triplicate injections.
*assign as enantiomeric impurity.
**mixture of (*RS*)-amlodipine and (*S*)-amlodipine at a final concentration (2:1).
